# Exploring student perceptions of the Osmosis digital learning platform in undergraduate medical education and its influences on motivation and inclusivity

**DOI:** 10.1186/s12909-025-07591-z

**Published:** 2025-07-11

**Authors:** Sanat Kulkarni, Erin Lawson-Smith, Laura Mongan, Rachel Westacott, Dawn Jackson

**Affiliations:** College of Medicine and Health, University of Birmingham, Birmingham, B15 2TT UK

**Keywords:** E-learning, Digital learning, Online learning, Learning motivation, Medical education, Self-determination theory, Self-efficacy, Qualitative research

## Abstract

**Background:**

The increasing incorporation of digital learning platforms has transformed pedagogical approaches in medical education. However, these tools are under-researched and under-theorised. In the 2022/23 academic year, an asynchronous, personalised digital learning tool (Osmosis) was provided to all medical students at one of the largest medical schools in the United Kingdom. We explored students’ experience of digital learning platforms, including Osmosis, and their influences on student motivation and inclusion.

**Methods:**

We conducted a qualitative study of second to final year medical students at the University of Birmingham. Data were collected through 10 semi-structured interviews and one focus group. The fifteen participants were purposively sampled based on year group, gender, ethnicity, international student status, self-reported health challenges, caring responsibilities and financial difficulties. Interviews were audio-recorded, transcribed and thematically analysed using the Framework Method. Data were analysed through the lens of self-determination theory (SDT) which focuses on the psychological needs of autonomy, self-efficacy and interconnectedness for achieving intrinsic motivation.

**Results:**

Five overarching themes were identified in relation to digital learning tools: (1) navigating complexity within the digital learning environment, (2) benefits and pitfalls of autonomous digital learning, (3) efficiency and depth in learning and promoting self-confidence, (4) social influences on digital learning and (5) curriculum level considerations. Aligned to SDT, digital tools promoted students’ autonomy, self-efficacy and relatedness thereby facilitating intrinsic motivation. Online tools, including the Osmosis platform, supported student inclusivity and accessibility, helping students overcome health and learning challenges. However, lack of formal guidance towards online platforms, misalignment to the local curriculum and concerns over reliability were key barriers to their use.

**Conclusion:**

Digital learning tools play an increasingly important role within modern medical education, positively impacting student motivation and inclusion. Nevertheless, greater focus must be placed on providing local guidance in accessing such tools, alongside constructively aligning their integration with other elements of the curriculum. Retaining medical students is vital to the future of patient care and it is ever more critical that higher education institutions prioritise student motivation, inclusion and wellbeing. Optimising integration of digital learning platforms therefore may be one means of achieving this.

**Clinical trial number:**

Not applicable.

**Supplementary Information:**

The online version contains supplementary material available at 10.1186/s12909-025-07591-z.

## Background

Digital learning tools are used increasingly within medical education but remain under-researched and under-theorised [1, 2]. In many medical schools, this technology is frequently used alongside traditional pedagogical approaches, such as in-person lectures and small group teaching. The accelerated implementation of digital approaches due to the COVID-19 pandemic has led to a paradigm shift in the delivery of medical education, creating an opportunity for medical schools to introduce previously novel tools [3]. This shifting educational environment has shaped how students access and gain knowledge and their interactions with one another [4]. 

Digital or e-learning can take many forms including online videos, textbooks, flashcards and question banks [4–6]. Digital learning platforms, defined here as web-based or computer-based software which deliver these educational materials [7], may be used synchronously by students at the same time, or asynchronously whereby students can access content at their own convenience [8, 9]. Asynchronous tools therefore offer flexibility in learning, tailored to the user, whilst simultaneously connecting students to a wider online community. Nevertheless, implementing digital learning platforms risks issues such as inaccessibility and an overwhelming volume of content [10, 11], which may limit their effectiveness [12, 13]. 

Current literature has shown emerging evidence of the efficacy of digital learning platforms with several studies highlighting the acceptability and benefits of an asynchronous approach [14–17]. Quantitative evidence from Malaysian and Japanese medical schools suggests that use of digital learning platforms is associated with greater learning motivation [18, 19]. However, existing studies often lack a theoretical underpinning leaving important gaps in our understanding of *how* these tools aid learning and motivation [17]. 

Undertaking theory-driven qualitative research enables deeper exploration of students’ digital learning experiences and priorities. By optimising digital approaches to meet student needs, medical schools can facilitate autonomous learning and motivation [20], providing the bedrock for better engagement, wellbeing [21] and academic performance [22]. 

Moreover, such research enables us to explore matters such as inclusivity and access in education. Community and connectedness may be fostered by digital learning tools thereby influencing educational equality [23]. On the other hand, a shift away from classrooms and towards virtual learning may risk social isolation, particularly amongst students who may reside at their family home away from other students [24, 25]. In the current climate, retaining medical students is a priority within our healthcare system. Engaging students’ needs to promote inclusivity and wellbeing is therefore critical, and digital learning tools may offer a solution, or even exacerbate underlying issues.

### Local context

This research initially focused on the Osmosis digital learning platform [26]. From 2022, full institutional access was given to all University of Birmingham medical students, providing them with access to its library of healthcare education videos, flashcards and question bank. The institutional license was paid for by the university enabling unlimited free access for students. The United States-created online platform was implemented as an asynchronous tool to use alongside the University’s virtual learning environment (Canvas) which acts as an online repository for curriculum and assessment information. Osmosis is an adaptive learning platform which guides students according to their learning needs, further promoting autonomous and flexible learning [27]. The mainstay of the platform are the short and accessible videos, often around 5–10 min in length, which summarise a topic or concept using simple ‘cartoons’, animations and diagrams. Positive feedback of Osmosis videos has been noted within the literature regarding their brevity, accessibility and engaging visuals compared to other online teaching methods [3, 28] but deeper exploration of the student experience through qualitative research is lacking.

### Theoretical frameworks

Students’ experiences of digital learning platforms, in particular Osmosis, and their influence on motivation and inclusivity was primarily explored through the lens of self-determination theory (SDT). Within SDT, motivation can be classified as extrinsic or intrinsic [29] with the latter associated with better performance and wellbeing [30, 31]. For intrinsic motivation to be maintained, three innate psychological needs must be met: (1) autonomy, (2) competence or self-efficacy and (3) relatedness to others. In exploring the digital learning environment, it is also important to consider connectivism which conceptualises knowledge as dynamic and flowing between nodes of learning communities, facilitated by technology [32]. Finally, as a research team, we have adopted an overarching critical educational stance which seeks to promote inclusion and empowerment of marginalised student groups [33]. Together these theories promote inclusivity, liberation and autonomous learning and therefore underpin the rationale and methodology of this study.

### AIMS

Through our theoretical lens, we sought to explore the following research questions:

1. How do digital learning tools, in particular Osmosis, influence student motivation?
2. How do digital learning tools, in particular Osmosis, influence student inclusion?

## Methods

### Study design and population

This is a single-centre study of second to sixth year medical students at the second largest medical school in the UK with approximately 400 students in each cohort [34]. Our study population therefore includes all students with access to Osmosis in the preceding 2022/23 academic year. There were no other specified inclusion or exclusion criteria. In May 2024, eligible students were recruited to the study via emails from the medical school and digital posters advertised during lectures, and students were invited to express their interest in sharing their experiences of digital learning tools and Osmosis in particular.

### Sampling

Interested students were presented with a short survey to facilitate purposive sampling (supplementary materials). Aligned to our critical stance and promotion of autonomy and liberty, all elements of the survey were optional, and students were asked to select their preference of interview medium. Options included an in-person or virtual individual interview or focus group, telephone call or providing a written response. Participants were purposively sampled across different groups (Table 1) to hear voices across the range of student experience, including those who may have faced barriers to engagement with some methods of traditional or digital learning. In alignment with our inclusive focus, these groups were sampled to reflect the diversity of our student cohort. Following sampling, students were invited to interview via e-mail according to their preferred choice of medium.

**Table 1:** Sampling approach for purposive sampling

STUDENT GROUP
o Gender
o Year of study
o Self-reported disability or health need that impacts their ability to interact with the MBChB course (for example a Reasonable Adjustment Plan but without requirement to detail the nature of this)
o Self-reported ethnicity
o Students who commute from their family home to either medical school or clinical placements
o Students who have experienced financial difficulties during the course of their studies (such as receipt of help from the Student Support Fund but without requirement to detail the nature of this)
o Students with caring responsibilities
o International students

### Data collection

In May and June 2024, data were collected through semi-structured interviews (in-person and virtual) and a facilitated focus group, providing within methods triangulation [35]. Distinct topic guides (supplementary materials) were piloted and used for the individual interviews and focus group, aligned to our study aims and overarching theoretical framework. These were not amended during the data collection phase. Whilst our initial focus was the Osmosis platform, it became apparent that participants’ perceptions of Osmosis, and digital learning more broadly, are embedded within a much wider ecosystem of online tools. We therefore expanded our inquiry to explore students’ experiences with other online learning tools, shining a light on the underworld of digital medical education. As an under-theorised area, we intended to explore the diverse reality of student engagement with digital tools, which includes instances where their choices diverge from resources provided by the medical school. While Osmosis served as our entry point, our interviews extended to other platforms, enabling understanding of the features valued most by students and therefore guiding the development or adoption of digital resources that are both accessible and truly embraced in practice.

Written informed consent was obtained prior to all interviews. All interviews were audio-recorded or video-recorded and transcribed verbatim, following which they were anonymised and checked for accuracy by the research team.

Data on Osmosis usage during the 2023/24 and 2024/25 academic years were collected directly from the online platform to provide insights into frequency and other patterns of use. This included data on the number of users stratified by year group and usage in the month prior to and during assessment periods. Data were also collected on use of different features within Osmosis, including the number of videos watched, number of questions answered, and flashcards used.

### Data analysis

**Fig. 1:**
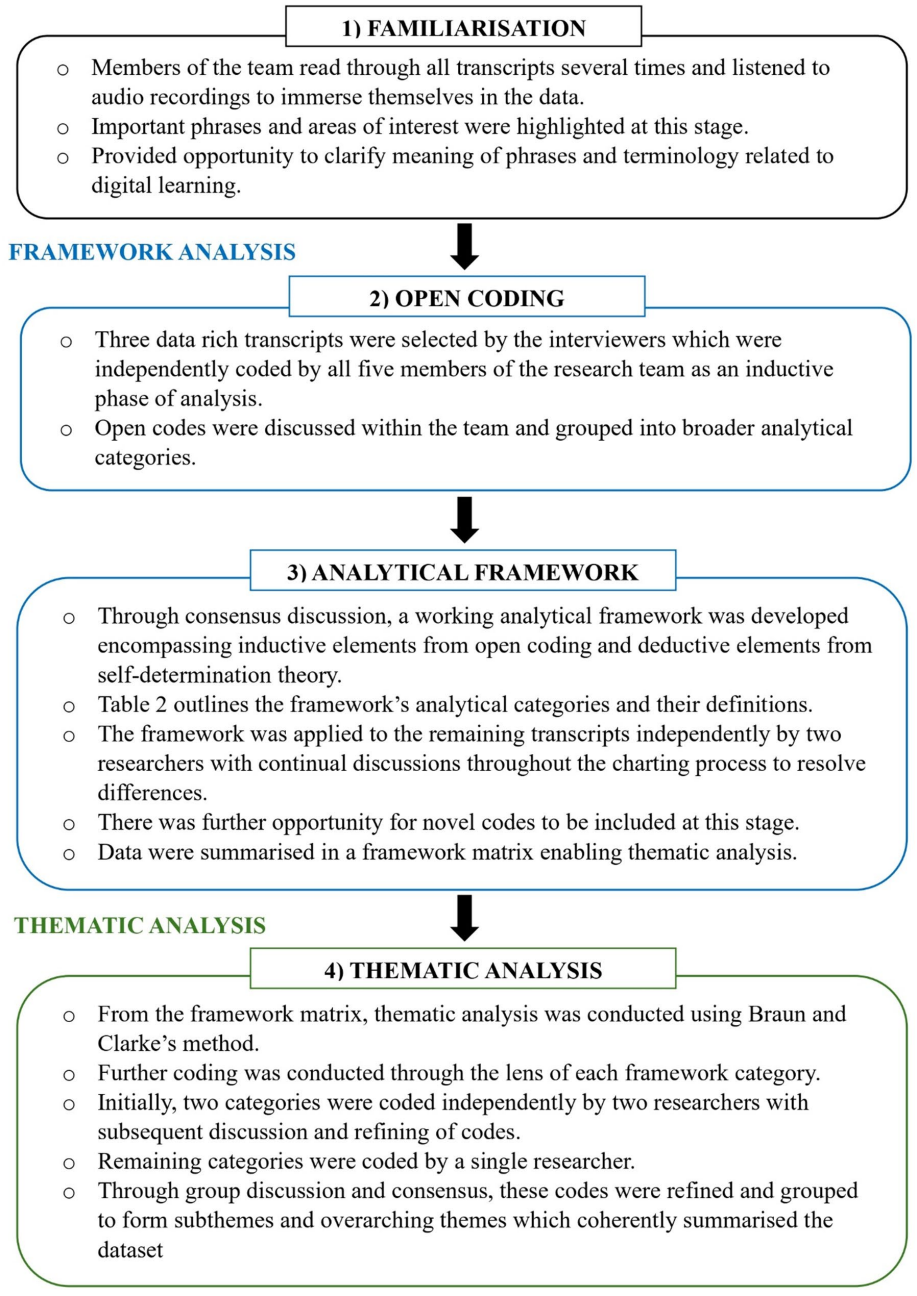
Framework Method of data analysis

Data were analysed using the Framework Method [36–38] originally conceived by Ritchie and Lewis [37], as outlined in Fig. 1; Table 2.

**Table 2:** Analytic framework categories and definitions

Category	Definition
***Autonomy***	Students in control of their learning, individualised, flexible approach
***Self-efficacy***	Student confidence and belief in the effectiveness of their learning approach
***Relatedness***	Learning with others, connected to messages and information about learning
***Facilitators***	Factors that have enabled engagement with digital learning, or positive results of digital learning. This also relates to elements such as trust and influencing factors.
***Barriers***	Factors that have hindered engagement with digital learning, or negative experiences/results of digital learning. This also relates to elements such as mistrust/unease and influencing factors

### Ethical approval

This study received ethical approval from the University of Birmingham Science, Technology, Engineering and Mathematics Committee (*ERN_0677*).

### Reflexivity

Regardless of the formal endeavours of medical schools, medical students typically also choose to supplement their learning using a range of digital tools to support their learning, which range in their cost, effectiveness and applicability [39, 40]. Without careful exploration, we risk a situation where students engage and purchase these tools based on ‘word of mouth’, without awareness of their limitations, applicability to learning objectives or alignment with effective learning strategies. As the second largest medical school in the United Kingdom, with a significant proportion of students from widening participation backgrounds, we wished to ensure that our learners had fair access to digital tools, that can provide benefits for all learners, regardless of their financial situation. In addition, we recognised the potential benefits of digital learning tools for students who may face barriers to engagement with ‘traditional’ learning methods. The Osmosis tool was introduced at the university in an attempt to provide a free-to-access, asynchronous learning tool, with a range of content and options for engagement. Accessibility was key, and the university paid for every medical student to have access to this tool. As with any educational intervention, it was important to the institution that this investment was providing a quality educational experience across our range of learners, and that it was being implemented effectively. Two members of the research team (RW and LM) were involved in the introduction and implementation of the Osmosis tool at institutional level, and provided key insights to its intended benefits, potential limitations and operational processes. Another researcher (DJ), experienced in qualitative research in medical education, provided guidance and supervision for the research design and thematic analysis. DJ was not involved in operational or strategic decisions around Osmosis implementation but has experience of implementing the tool as a senior educator for medical students in their clinical years. RW, LM and DJ were recognised to have leadership roles in the undergraduate medicine programme, and were not involved in the data collection with students, who may have felt unable to share openly about negative experiences of digital learning, or their choices to deviate from intended use. SK (a clinical teaching fellow and resident doctor) and EL-S (a recently-qualified resident doctor) undertook all interviews. As a teaching fellow, SK had previously taught several participants although without specific use of digital tools such as Osmosis. No participants were known to EL-S. All research team members were involved in data analysis and met regularly to develop and refine the framework analysis matrix. SK performed analysis of all interviews, and DJ, LM, RW and E-LS worked together with SK, through analytical cycles, resolving disagreement through discussion and consensus. DJ provided oversight and support for the approach to analysis for all staff. SK, DJ and LM developed and refined the presented themes.

## Results

### Osmosis usage

Data on Osmosis usage in the 2023/24 and 2024/25 academic years are summarised below in Table 3. As demonstrated, use was highest amongst first year students (14.8% of students per week on average in 2024/24) and lowest amongst fifth year students (4.8% of students per week on average in 2024/25). Use of the platform appeared to be consistent throughout the year amongst students with only small observed increases in activity during assessment periods. Usage was broadly similar across both academic years evaluated. Videos were the most used feature within the platform; the question bank and flashcard features were infrequently used in comparison.

**Table 3:** Osmosis usage during the 2023/24 and 2024/25 academic years

Year group	Total number of students		Median number of Osmosis users per week (%)		Median number of users per week during and 4 weeks prior to January assessment period (*N*%)		Median number of users per week during and 4 weeks prior to April/May assessment period (*N*%)		Median number of Osmosis videos watched per week (maximum)		Median number of Osmosis questions answered per week (maximum)		Median number of completed Osmosis flashcards per week (maximum)	
Academic year	23/24	24/25	23/24	24/25	23/24	24/25	23/24	24/25	23/24	24/25	23/24	24/25	23/24	24/25
**1**	397	450	51 (12.8)	66.5 (14.8)	109 (27.5)	111 (24.7)	83 (20.9)	58 (12.9)	61 (333)	107 (465)	47 (345)	104 (345)	84 (876)	64 (3226)
**2**	361	377	60.5 (16.8)	38.5 (10.2)	111 (30.7)	56 (14.9)	95 (26.3)	44 (11.7)	61.5 (461)	53.5 (288)	33 (236)	14 (113)	34 (302)	5 (569)
**3**	409	356	53.5 (13.1)	39.5 (11.1)	N/A	N/A	67 (16.4)	53 (14.9)	47.5 (187)	56 (235)	13 (195)	12 (84)	18 (445)	2.5 (149)
**4**	446	448	44 (9.9)	50 (11.2)	N/A	N/A	63 (14.1)	46 (10.3)	54.5 (164)	69 (146)	97.5 (530)	14 (202)	24 (123)	24 (123)
**5**	433	442	15 (3.5)	21 (4.8)	N/A	N/A	22 (5.1)	27 (6.1)	22.5 (64)	83 (130)	85 (330)	592.5 (1342)	168 (638)	0 (39)

### Participants

Ten individual semi-structured interviews and one facilitated focus group (*n* = 5) were conducted with a total of 15 participants. The participant characteristics are outlined in Table 4. Participants were purposively sampled across all intended categories. The median interview duration was 43 min (range 32–73 min). Due to the deductive and inductive approaches to framework analysis, data saturation was considered to be the point where predetermined categories were adequately represented in the data, and also the extent to which no further novel categories were identified within the data. Data saturation was reached at 12 interviews.

**Table 4:** Participant characteristics

Participant number	Year of study	Gender	Ethnicity	Sampled characteristics	Type of interview	Interview duration (mins.)
1	3	Male	Arab	Commuter	Focus group	73
2	3	Female	Asian or Asian British - Pakistani	Health/learning challenges, carer, commuter	Virtual interview	54
3	2	Male	Chinese	N/A	Interview	55
4	4	Female	White British	Health/learning challenges	Virtual interview	40
5	3	Female	White British	Health/learning challenges, financial support	Virtual interview	54
6	3	Female	White British	N/A	Focus group	73
7	3	Male	Asian or Asian British - Bangladeshi	N/A	Interview	37
8	3	Male	Other White background	Health/learning challenges	Virtual interview	58
9	4	Male	Asian or Asian British - Indian	Health/learning challenges	Virtual interview	43
10	4	Female	Mixed– White and Asian	N/A	Virtual interview	33
11	4	Male	White British	N/A	Virtual interview	41
12	3	Male	Asian or Asian British– Indian	International student	Focus group	73
13	3	Female	Chinese	International student	Focus group	73
14	5	Female	Asian or Asian British - Pakistani	Commuter	Focus group	73
15	5	Female	Arab	N/A	Virtual interview	32

## Themes

Through our framework and thematic analysis, five overarching themes with underlying subthemes were identified, as summarised in Table 5.

**Table 5:** Themes and subthemes

THEME	SUBTHEME
**Navigating complexity within the digital learning environment**	Digital resource variability and overload
	Lack of clarity and support
**Benefits and pitfalls of autonomous digital learning**	Enabling choice
	Challenges of self-directed learning
**Efficiency and depth in learning and promoting self-confidence**	Streamlined digital learning processes
	Enhancing understanding and confidence
**Social influences on digital learning**	Guidance towards resources
	Enhancing connectedness through anonymity
	Challenges in collaborative digital learning
**Curriculum level considerations**	Curriculum alignment
	Clinical application
	Trust and reliability

### Theme 1: navigating complexity within the digital learning environment

#### Subtheme: digital resource variability and overload

Within the digital sphere there was a perceived saturation of available resources coupled with variability in their perceived quality.

*“It’s quite saturated already with the amount of* [digital resources] *we have.”*– Participant 9.

*“I’d say they’re* [online platforms] *not always the most consistent*,* so one topic might be covered really well*,* but then something else is really short and that’s probably not enough for what I need to know.”*– Participant 10.

#### Subtheme: perceived lack of clarity and support

Students expressed frustration at the lack of clarity regarding the expected standard of knowledge, subsequently limiting integration and alignment with the Osmosis platform.

*“The medical school weren’t very clear about what we need to know and what was additional information.”*– Participant 2.

*“Occasionally when we’re directed to Osmosis through a Canvas module*,* it will be five or six different links to different Osmosis videos for one topic that they’re trying to cover. That’s quite overwhelming. You think about targeting all these things to be able to cover one learning objective.”*– Participant 14.

Further exacerbating these challenges was a perceived lack of guidance from the medical school towards utilising digital tools, coupled with dissonance between the students and faculty regarding their perceived utility.

*“It seems like the medical school tell you not to* [use digital learning tools]. *And everyone basically ignores the medical school and does their own thing… I do really think the biggest thing would just be if we could be told a bit more about how best to use them and actually encouraged to use them from an earlier stage… it feels like you’ve got the medical school at one complete end of the spectrum and all the students on the other end and everyone’s just sort of lying to each other.”*– Participant 11.

### Theme 2: benefits and pitfalls of autonomous digital learning

#### Subtheme: enabling choice

The wide variety of digital learning tools offered students choice over the content and depth of their learning, fostering empowerment and motivation.

*“Digital learning allows you to prioritise the things you want to more than having a teaching session on certain topics every day”.*– Participant 9.

*“If I’m on PassMedicine and there’s a bit on the epidemiology of diabetes*,* I’m just going to skip over that because I’m just not too interested. Whereas if you’re sitting in a session… they’re going to spend 5 minutes talking about the epidemiology”*– Participant 11.

*“I think I’d mainly start by using PassMedicine*,* Quesmed or BiteMedicine just to make notes on each condition*,* such as pathophysiology*,* investigations*,* management*,* diagnosis. If there was something I didn’t understand*,* then I would look more into it with using YouTube videos by doctors or Osmosis to understand concepts more than just get content.”*– Participant 10.

Moreover, the choice enabled students to use resources which they found most helpful, for example using those with dual representation as visual elements to aid their learning.

*“So everyone works differently. When I spoke to some of my colleagues about how they learnt*,* some of them went straight on to questions and that’s their mode of learning. Whereas for me*,* I need to understand how things work… I need to read up about a condition first”*– Participant 2.

Digital learning tools also enabled flexibility and convenience along with control over pace of learning.

*“I coach netball a lot and I go to my sister’s netball games a lot and I could sit there in the break beforehand*,* or if my team wasn’t playing*,* I could sit there on my phone and do a quick 10 questions”*– Participant 5.

In addition, some students felt this variety and choice in digital resources improved accessibility and inclusivity for students with health challenges.

*“I spent all the first year and most of the second year with impaired hearing. So anytime a lecturer had a strong accent or subtitles weren’t done*,* it was another barrier… Accessibility was so much better with Osmosis and PassMedicine… Osmosis you can put subtitles on so that was great.”*– Participant 5.

#### Subtheme: challenges of self-directed learning

Students expressed that the challenge of managing their own learning contributed to a sense of feeling overwhelmed.

*“So pacing is difficult when you’re doing self-studying*,* but you still have to deal with how the university is pacing everything.”*– Participant 3.

This was exacerbated by the perception that the medical school failed to adapt to different learning preferences, limiting autonomous learning.

*“The medical school don’t support well in terms of outside learning… the different learning styles aren’t adapted to”*– Participant 5.

### Theme 3: efficiency and depth in learning and promoting self-confidence

#### Subtheme: streamlined digital learning processes

Students highly valued the organisation, ease of access and efficiency that digital learning tools offered, which fostered their engagement and motivation. A common example was the ability to “*speed up*” or skip parts of videos. Given the volume of information to learn, students valued the organisation and efficiency offered by digital tools.

*“I don’t want to use the word cutting corners because I feel like that makes it sound like we’re trying to like find the easier way*,* but it’s not. We’re trying to get to the information that we need as fast as possible*,* and sometimes I feel like there’s a little bit of a disconnect where the Medical school don’t want us to only have the information that we absolutely need*,* because that doesn’t enrich our learning or whatever*,* but we don’t have the time because there’s so much demand. Whereas on these other places it’s got everything we need… super easy”*– Participant 9..

#### Subtheme: enhancing understanding and confidence

Many students commented that Osmosis helped to provide deeper understanding of the material which in turn fuelled intrinsic motivation to learn.

*“I noticed that things that I’d seen in one Osmosis video I was able to apply to another concept because I was able to make links and understood that a certain process happens here and this is what’s happening here*,* which explains what I’m learning. And that reasoning helped me to make links and understand things better.”*– Participant 2.

*“I don’t want my knowledge to become pattern recognition where I can just answer a question. I’d like to know about the condition deeper.”*– Participant 11.

The use of digital learning tools promoted student confidence over time, leading to a shift away from prescribed medical school content between the pre-clinical and clinical years. Across participant accounts, clinical phase students invariably utilised digital platforms in some capacity.

*“I used the free trial* [for Osmosis]. *I noticed that it was really useful because it was really increasing my knowledge and I was starting to understand things a lot better and I could see the impact that it was having on my own learning.”*– Participant 2.

*“In pre-clinical*,* I mainly did the University lectures… But then for clinical I have not used any of the lectures… I’ve only used PassMedicine*,* Quesmed*,* BiteMedicine and then Osmosis.”*– Participant 10.

### Theme 4: social influences on digital learning

#### Subtheme: guidance towards resources

The overwhelming majority of participants highlighted the role of peers, including those in the same year and older students, in guiding them towards online resources. This was largely through ‘word of mouth’ discussions and, to a lesser extent, awareness of others’ use of digital tools. In several circumstances, this included use of digital tools created by other students.

*“Definitely word of mouth is a big thing. When you hear from older years about what they’ve used and they’ve been successful within the past*,* you tend to think that’s worked for them*,* that will probably work for me because the content is the same.”*– Participant 9.

#### Subtheme: enhancing connectedness through anonymity

Students also acknowledged the benefits of anonymous discussion forums and comments sections within digital platforms which contributed to their enjoyment of learning, motivation and removed the fear of embarrassment and failure. However, this feature is not included within the Osmosis platform but offered on alternative websites.

*“Sometimes if I answer an online question wrong then I’ll use the comments section to check and somebody else no doubt will have said a very similar thing. And then somebody explained the answer and… I love the fact that everyone helps everyone else out.”*– Participant 5.

*“You can be wrong in PassMedicine and no one is gonna know. You can be anonymous*,* there’s none of the same things to lose”*– Participant 4.

#### Subtheme: challenges in collaborative digital learning

However, whilst students benefitted from asynchronous and anonymous collaboration, many disliked the use of such tools in a synchronous group setting as it limited their autonomy and efficiency.

*“It’s about time efficiency.* [Doing questions in groups is] *not the best*,* because you also want to get to the stuff you find difficult. So if you’re spending 5 minutes talking through a question someone else finds difficult*,* it’s not always the best.”*– Participant 11.

### Theme 5: curriculum level considerations

#### Subtheme: curriculum alignment

Particularly with the Osmosis platform, a common cause for concern was the misalignment between the platform’s more United States (US)-focused content and the UK curriculum.

*“Sometimes I don’t fully rely on it because it’s American… I don’t know that all their management things are UK based.”*– Participant 10.

#### Subtheme: clinical applications

Similarly, this led to the common perception that Osmosis is less aligned to UK clinical practice and, by extension local assessments.

*“I would just take some of their things* [Osmosis] *with a pinch of salt… Just knowing that like our questions are all based on NICE guidelines and NHS things.”*– Participant 10.

In contrast, other UK-focused tools better bridged the gap between theoretical learning and clinical practice, further motivating students.

*“But the online resources that were available were just so convenient and so simple… what was nice is that I could see the knowledge I was learning actually being used. And on placement it was the same thing.”*– Participant 8.

#### Subtheme: trust and reliability

The reliability of digital learning resources was a key factor in students’ choice of tool with preference given to ‘official’ or trustworthy sources, rather than other online videos or student-made resources.

*“I’m likely to trust it because I know that Osmosis is done by Elsevier*,* which are the company that do the textbooks.”*– Participant 7.

*“It’s the accuracy of the information and the answers… that reliability is a big thing.”*– Participant 13.

*“I wouldn’t go to just any YouTube video and look for a condition. I’d look for a verified account…”*– Participant 9.

## Discussion

### Summary of key findings

This study explored medical students’ experiences of digital learning tools through the lens of self-determination theory. It highlights the challenges faced by new medical students who must navigate the large volume of traditional and online resources available to them, often with perceived paucity of faculty support. As they progress through medical school, students became increasingly autonomous in their learning, facilitated by the wide variety of digital learning tools. This research also demonstrated further advantages of digital learning tools including their efficiency, accessibility of information and ability to instil deeper understanding and self-confidence. Moreover, digital learning platforms fostered interconnectedness and collaboration between peers whilst also providing a means of overcoming health and learning challenges. Beyond nurturing inclusivity, the tools’ direct applications to clinical practice and perceived effectiveness promoted intrinsic motivation amongst students.

However, the research also illustrates several barriers to the implementation of digital learning platforms in medical curricula. Encouraging autonomous learning creates a need for structured institutional support which at times was perceived as lacking by participants. Students sought greater guidance on accessing and best utilising digital platforms, along with more acknowledgement of their potential benefits in learning. Secondly, awareness of and direction towards such tools was reported primarily through informal networks rather than from the medical school itself. Finally, the perceived misalignment of tools to local curricula and concerns over reliability may limit their utility in a population heavily focused on assessments.

This constellation of factors may explain the relatively low observed usage of Osmosis across the cohorts, particularly amongst final year students who may have established learning strategies by that stage of study. Furthermore, the plethora of available digital learning platforms for medical students was apparent throughout the interviews. The UK-focus of platforms such as PassMedicine [41]Quesmed [42] and BiteMedicine [43] offered greater applicability to students’ assessments and future clinical practice than the US-centric Osmosis platform. These platforms offer extensive question banks alongside online written textbooks and recorded webinars. Unlike Osmosis, each of these platforms are specifically tailored to UK curricula with perhaps a greater focus on assessment preparation through multiple choice questions as opposed to Osmosis’ more content delivery focus through videos. This differing focus is reflected within our quantitative data in which videos were the most commonly used feature within Osmosis, whilst our participants typically preferred use of UK-centric platforms for assessment preparation.

In a similar fashion to Osmosis, these UK-based platforms also encouraged autonomous learning through their asynchronous design, allowing students to access learning in their own time and receive instantaneous feedback. Moreover, the anonymous discussion feature within the PassMedicine platform was frequently appreciated by students, fostering a sense of interconnectedness. Anonymity removed students’ fear of failure, increasing their self-efficacy, confidence and enjoyment; this feature is currently unavailable within Osmosis. Coupled with signposting towards these established UK platforms from trusted peer networks, it is perhaps unsurprising that they outcompeted the Osmosis platform for participants’ time.

Nevertheless, those using the Osmosis tool felt it fostered greater understanding of topics, allowing students to form links with other areas of the curriculum and in turn instilling self-confidence. Moreover, the use of subtitles within videos and asynchronous nature of the platform enhanced accessibility and inclusivity, even helping one student overcome a specific health challenge. Based on its institutional associations, students placed trust in the Osmosis platform.

### Comparison with existing literature and theoretical frameworks

Our findings support those observed in previous studies exploring asynchronous digital learning tools. In particular, the flexibility, convenience and personalised nature of asynchronous digital learning is universally acknowledged across studies [10, 14, 16, 17]. Similarly, the risks of overwhelming students [10], importance of usability [14] and clinical relevance of the tools [44] align with prior research and educational theory [45]. In contrast, technical barriers such as digital competency were rarely raised by our participants, reflective of the assimilation of technology in modern education.

Building on this literature, our research highlights how digital learning tools can promote student motivation and learning, in alignment with self-determination theory [29]. As outlined above, digital learning tools aid autonomous learning in terms of content, pace and depth whilst promoting self-efficacy and confidence through instilling deeper understanding of material. In contrast however, the vast array of digital learning tools appeared to negatively impact students’ self-efficacy, with reports of feeling overwhelmed with difficulties in navigating digital tools. Moreover, it appeared that motivation was often context-specific and dependent on curriculum alignment, highlighting the importance of external factors, and the role of faculty and curriculum design. Whilst there are promising signs for the role of digital tools in fostering student motivation, there are equally several threats to their successful implementation. Medical schools can potentially mitigate these through careful curriculum planning and student support.

When considering connectivism [32], our research elucidates several social factors influencing access to the digital learning environment and its role in facilitating the flow of knowledge between learners. On one hand, digital tools can break down barriers between learners, facilitated by anonymity, thereby stimulating discussion and transmission of knowledge across student groups. Building on this further, tools also enabled students to overcome important health challenges, thus further encouraging both their engagement with course material and greater interconnectedness with the student cohort. While our study highlighted the benefits of virtual interconnectedness, guidance on the use of digital tools primarily came from near-peer recommendations, underscoring the significance of the informal curriculum [46]. Aligned to connectivism and the need for relatedness within SDT, our results highlight the role of social relationships and exchanges in governing use of educational resources. In turn, this may lead to a digital divide in utilising online tools between more connected and marginalised student groups and therefore risks variable experience between students [47]. Moreover, synchronous use of digital platforms risked compromising students’ autonomy and self-efficacy, highlighting the conflicts between the psychological needs within SDT [29]. 

### Implications and recommendations

Improved signposting to digital platforms, along with support and guidance from the medical school may mitigate the risk of a ‘digital divide’ between those students who are well-connected, and those who are not. This early support for autonomous learning may also allay concerns over students feeling lost and overwhelmed at the start of the course. As students develop personalised learning strategies over the course, the focus must instead shift to providing support in integrating digital learning with clinical placements. With almost ubiquitous use of digital platforms in the clinical years, medical schools must consider ways to optimally balance digital and experiential learning.

Furthermore, the lack of perceived relevance of the Osmosis platform to UK medical school curricula and assessment highlights the need to consider constructive alignment when integrating asynchronous digital learning tools. Constructive alignment ensures that students’ learning activities relate to their prescribed outcomes and, by extension, their assessments [48]. Moving forward, raising awareness of such platforms amongst faculty and content mapping to specific learning outcomes is vital. Doing so will not only aid integration of the platform with existing resources but can also highlight gaps across the traditional and digital modalities. Particularly considering digital platforms with broad content coverage, curriculum mapping can enable better organisation and streamlining of content thus meeting student demands for efficiency [49]. Additionally, mapping creates an opportunity to verify the quality and accuracy of the online learning materials, which, although resource intensive, is vital for the success of any e-learning intervention [50]. 

Finally, medical schools should place inclusivity and wellbeing at the forefront of curriculum design [1]. As demonstrated within our study, digital learning tools can mitigate the impacts of health and learning challenges through offering flexible working schedules, addressing accessibility requirements and encouraging anonymous discussions. These lessons underline the importance of accurate subtitles and lecture transcripts in faculty-developed resources and the need to embrace anonymity within the virtual learning environment.

### Strengths and limitations

This theory-driven qualitative study included a diverse range of participants from a variety of sociodemographic backgrounds and at differing stages of their medical school journey. This purposive approach enhances the depth and relevance of our findings regarding digital learning experiences. Moreover, the use of a systematic and rigorous analytic approach, embedded within a theoretical framework, enabled comparisons both within and between participant accounts with triangulation between individual interviews and the focus group [36]. 

However, by nature of the recruitment and sampling process, it is plausible that students with stronger views concerning Osmosis and digital platforms were more likely to participate. We were unable to link individual Osmosis usage data to specific participants which would have empirically quantified their engagement and specific features used. In addition, the timing of data collection immediately following the assessment period may have led to examination results positively or negatively influencing their perception of the tools. Moreover, this window for data collection would have significantly reduced the likelihood of students who were resitting assessments opting to participate; the experiences of this group will be important to explore moving forward. The single-centre nature of this study may limit its external validity, particularly given the importance placed on local factors, such as medical school support and curriculum design, by our study participants.

### Directions for future research

Whilst our findings begin to paint a picture of students’ experiences of digital learning tools, larger scale, theory-driven quantitative research can capture the perspectives of the wider student population beyond a single university. Our findings may guide survey development, but additional use of validated self-determination theory scales can quantify the impact of digital learning tools on student motivation. In turn, these measures can be correlated with students’ academic performance to provide further justification for implementing digital learning platforms. Furthermore, our research focuses solely on the student voice; exploring the perspectives of faculty as another key stakeholder is an important next step.

## Conclusion

Digital learning tools, including the Osmosis platform, can foster motivation and inclusivity amongst undergraduate medical students. Aligned to self-determination theory, these tools meet students’ psychological needs for autonomy, self-efficacy and relatedness thereby facilitating intrinsic motivation. Nevertheless, a perceived lack of medical school guidance and support and, particularly with the Osmosis platform, misalignment with the local curriculum were notable barriers to their use.

## Electronic supplementary material

Below is the link to the electronic supplementary material.

Supplementary Material 1

## Data Availability

The datasets used and/or analysed during the current study are available from the corresponding author on reasonable request.
