# Supplementary material for: Exploring student perceptions of the Osmosis digital learning platform in undergraduate medical education and its influences on motivation and inclusivity
Source: BMC Med Educ. 2025 Jul 11;25:1041. doi: 10.1186/s12909-025-07591-z (PMC12255063; doi:10.1186/s12909-025-07591-z)
Supplement: Supplementary file 1 — Supplementary Material 1 [file 12909_2025_7591_MOESM1_ESM.docx]

## **SUPPLEMENTARY MATERIALS**

**Supplementary Materials 1 (S1)**: Sampling survey

**Supplementary Materials 2 (S2)**: Semi-structured topic guide used in (A) facilitated focus groups and (B) interviews

**Supplementary Materials (S1)**: *Sampling survey*

**Medical student experiences of online learning and Osmosis (administered via Microsoft Forms)**

Prefacing statement: This project aims to understand medical student experiences of digital learning and the Osmosis digital learning platform. We plan to interview medical students. This short survey will help us with our sampling strategy and to collect information on your preferred interview format and date/time. You can access the participant information sheet for the survey here: (embedded link to the participant information sheet)

* = required response

**SECTION 1**

**1. If you’d like to take part in our interviews/focus groups, please provide your email so that we can arrange a slot with you.** *

**2. Please indicate your preferred interview format(s) (please tick all that apply)**

A: 1:1 interview (virtual, video-call on MS Teams)

B: 1:1 interview (face-to-face, at Medical School)

C: Focus group interview (virtual, on MS Teams)

D: Focus group interview (face-to-face, at Medical School)

E: 1:1 interview (virtual, telephone call on MS Teams)

F: I would prefer to provide a written response

**3. Please indicate the timeslots that you would be available to conduct your interview.**

A: Dates/times to be inserted here when confirmed

**SECTION 2**

**We would also like to collect some additional information about you. This information is OPTIONAL but would help us to ensure we capture a wide range of views.**

**4. Please indicate your year of study on the MBChB course (optional)**

A: Year 2

B: Year 3

C: Year 4

D: Year 5

**5. Please indicate your gender below (optional)**

A: Woman

B: Man

C: Non-binary

D: Prefer not to say

**6. Please indicate your ethnicity below (optional)**

A: White British

B: White Scottish

C: Irish Traveller

D: Gypsy or Traveller

E: Other White background

F: Black or Black British – Caribbean

G: Black or Black British – African

H: Other Black background

I: Asian or Asian British – Indian

J: Asian or Asian British – Pakistani

K: Asian or Asian British – Bangladeshi

L: Chinese

M: Other Asian background

N: Mixed – White and Black African

O: Mixed – White and Black Caribbean

P: Mixed – White and Asian

Q: Other Mixed background

R: Arab

S: Other

**7. Please indicate if you have experienced health or learning challenges during the study of undergraduate medicine (this may include the requirement of a Reasonable Adjustment Plan, but does not have to) (optional)**

A: Yes

B: No

C: Prefer not to say

**8. Please indicate if you have struggled financially during the study of undergraduate medicine (this may include the receipt of help from the Student Support Fund, but does not have to) (optional)**

A: Yes

B: No

C: Prefer not to say

**9. Please indicate if you have caring responsibilities alongside your undergraduate programme of study (optional)**

A: Yes

B: No

C: Prefer not to say

**10. Please indicate if you are a local or commuter student (this is if your home address is the same as your term time address) (optional)**

A: Yes

B: No

C: Prefer not to say

**11. Please indicate if you are an international student (optional)**

A: Yes

B: No

C: Prefer not to say

**Supplementary Materials 2 (S2)**: *Semi-structured topic guide used in* (A) *facilitated focus groups and* (B) *interviews*

**(A)**

| **Question number** | **Question** |
| --- | --- |
| **1)** | What have been your experiences of using the Osmosis platform?  *Prompt: has anyone else had similar or different experiences?* |
| **a)** | What has worked well with regards to the introduction of the Osmosis platform?  *Prompt: why has it worked well? Have others found this has worked well for them and if so, why?* |
| **b)** | What has not worked so well?  *Prompt: why has is not worked so well? Has anyone else had similar or not so similar experiences?* |
| **2)** | Does Osmosis play a role in learning alongside other university resources? Explain.  *Prompt: how do you think it could better complement university resources?* |
| **3)** | Why do you use Osmosis? If not, why not? |
| **a)** | Do you use any other online learning platforms?  *Prompt: if so, which ones and why?* |
| **4)** | Do you use Osmosis on your own or in a group setting?  *Prompt: do you find this useful or not?* |
| **a)** | Which features of Osmosis do you find useful (or not useful)? |
| **b)** | Are they any particular topics/areas for which Osmosis is particularly useful (or not useful)?  *Prompt: why in particular are these useful (or not useful)?* |
| **5)** | What changes would you like to see made at the University of Birmingham with regards to the Osmosis platform? |
| **6)** | Is there anything else you would like to share? |

**(B)**

| **Question number** | **Question** |
| --- | --- |
| **1)** |  |
| **a)** | During your time at medical school, what ways or approaches have you found to be useful for your learning?  *Prompt: why do you think have these been useful or worked well?* |
| **b)** | What ways or strategies have not worked so well?  *Prompt: why do you think these have not worked well?* |
| **c)** | Has the way (or ways) you learn changed over the course of university so far? If so, how?  *Prompt: why do you think it has changed? E.g. due to a shift from pre-clinical to clinical medicine?* |
| **d)** | What have been your experiences of learning in groups versus learning alone?  *Prompt: what has worked well and not so well in this regard?* |
| **2)** | What are your thoughts on digital learning tools? |
| **a)** | What are your thoughts on the Canvas platform? |
| **b)** | What have been your experiences of the Osmosis platform? What has worked well and what has not worked so well? |
